# Cicatrix Removed by Medicine

**Published:** 1884-06

**Authors:** J. T. Kent

**Affiliations:** St. Louis


### CLINICAL BUREAU.

---

### CICATRIX REMOVED BY MEDICINE.

Prof. J. T. Kent, A. M., M. D., St. Louis.

A young lady twenty-six years old, consulted me for some cicatrices on the left side of the neck. An indentation that disfigured her very much was there. She said with the exception of cold, damp feet, she was in good health. The fistulous openings had been three, discharging several years, and finally closed under some sort of blood, or root sirup. Believing that her treatment had only temporarily controlled the trouble, I attempted to find what her remedy should be. From all I could glean, and she had very few symptoms, but the Calc. c. symptom of "cold damp stockings" was there. She took one dose of Calc. c. 85m (Fincke.)

On the third day her neck began to be painful. She called to ask me if the medicine had anything to do with it. Plenty of S. L. was given. The deep cicatrix suppurated and discharged several calcareous nodules and the neck healed with scarce a scar where the one opened. A depression about two inches from this one is unsightly. She wishes that had opened in like manner. But a little surgical skill may remove the other.

Lippe gives, Cicatrices breaking open: Carbo v., Crocus, Crotal, *Lach.*, Nat. m., Phos., Sil.

